# Cyclotrons Operated for Nuclear Medicine and Radiopharmacy in the German Speaking D-A-CH Countries: An Update on Current Status and Trends

**DOI:** 10.3389/fnume.2022.850414

**Published:** 2022-04-14

**Authors:** Claus Zippel, Johannes Ermert, Marianne Patt, Franz Josef Gildehaus, Tobias L. Ross, Gerald Reischl, Torsten Kuwert, Christoph Solbach, Bernd Neumaier, Oliver Kiss, Markus Mitterhauser, Wolfgang Wadsak, Roger Schibli, Klaus Kopka

**Affiliations:** ^1^Professur für Betriebswirtschaftslehre und Management im Gesundheitswesen, KH Mainz, Mainz, Germany; ^2^Institute for Neuroscience and Medicine, INM-5: Nuclear Chemistry, Forschungszentrum Jülich, Jülich, Germany; ^3^Klinik und Poliklinik für Nuklearmedizin, Universität Leipzig, Leipzig, Germany; ^4^Klinik und Poliklinik für Nuklearmedizin, Ludwigs-Maximilians-Universität München, München, Germany; ^5^Klinik für Nuklearmedizin, Medizinische Hochschule Hannover, Hannover, Germany; ^6^Abteilung für Präklinische Bildgebung und Radiopharmazie, Universitätsklinikum Tübingen, Tübingen, Germany; ^7^Cluster of Excellence iFIT (EXC 2180) “Image Guided and Functionally Instructed Tumor Therapies”, University of Tübingen, Tübingen, Germany; ^8^Nuklearmedizinische Klinik, Universitätsklinikum Erlangen, Erlangen, Germany; ^9^Klinik für Nuklearmedizin, Universitätsklinikum Ulm, Ulm, Germany; ^10^Institute of Radiopharmaceutical Cancer Research, Helmholtz-Zentrum Dresden-Rossendorf, Dresden, Germany; ^11^Ludwig Boltzmann Institute Applied Diagnostics, Vienna, Austria; ^12^Division of Nuclear Medicine, Medical University of Vienna, Vienna, Austria; ^13^Department of Inorganic Chemistry, Faculty of Chemistry, University of Vienna, Vienna, Austria; ^14^Center for Radiopharmaceutical Sciences, Paul Scherrer Institute, Villigen, Switzerland; ^15^Faculty of Chemistry and Food Chemistry, School of Science, TU Dresden, Dresden, Germany

**Keywords:** (medical) cyclotron, radionuclide production, nuclear medicine, infrastructure for radiopharmaceutical production, radiation equipment and supplies

## Abstract

**Background:**

Cyclotrons form a central infrastructure and are a resource of medical radionuclides for the development of new radiotracers as well as the production and supply of clinically established radiopharmaceuticals for patient care in nuclear medicine.

**Aim:**

To provide an updated overview of the number and characteristics of cyclotrons that are currently in use within radiopharmaceutical sciences and for the development of radiopharmaceuticals to be used for patient care in Nuclear Medicine in Germany (D), Austria (A) and Switzerland (CH).

**Methods:**

Publicly available information on the cyclotron infrastructure was (i) consolidated and updated, (ii) supplemented by selective desktop research and, last but not least, (iii) validated by members of the committee of the academic “Working Group Radiochemistry and Radiopharmacy” (AGRR), consisting of radiochemists and radiopharmacists of the D-A-CH countries and belonging to the German Society of Nuclear Medicine (DGN), as well as the Radiopharmaceuticals Committee of the DGN.

**Results:**

In total, 42 cyclotrons were identified that are currently being operated for medical radionuclide production for imaging and therapy in Nuclear Medicine clinics, 32 of them in Germany, 4 in Austria and 6 in Switzerland. Two thirds of the cyclotrons reported (67%) are operated by universities, university hospitals or research institutions close to a university hospital, less by/in cooperation with industrial partners (29%) or a non-academic clinic/ PET-center (5%). Most of the cyclotrons (88%) are running with up to 18 MeV proton beams, which is sufficient for the production of the currently most common cyclotron-based radionuclides for PET imaging.

**Discussion:**

The data presented provide an academically-updated overview of the medical cyclotrons operated for the production of radiopharmaceuticals and their use in Nuclear Medicine in the D-A-CH countries. In this context, we discuss current developments and trends with a view to the cyclotron infrastructure in these countries, with a specific focus on organizational aspects.

## Background

**Figure 1:**
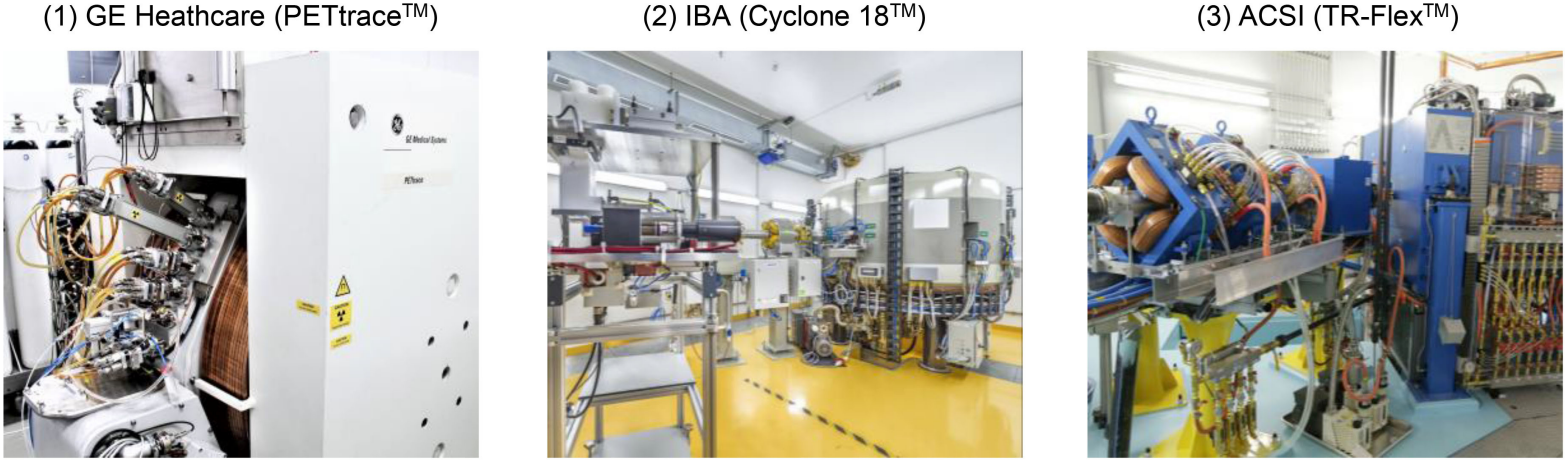
Examples of three medical cyclotrons operated in the D-A-CH region from different manufacturers. Photograph: (1) University Hospital Ulm/Solbach, (2) HZDR/Künzelmann, (3) HZDR/Bierstedt.

In recent years nuclear medicine has played an increasingly important role in many clinical areas and precision medicine, especially in oncology, neurology and cardiology (1, 2). A main driver of this development are innovative approaches in nuclear medicine imaging and therapy, which are based primarily on new (theranostic) radiotracer/radiopharmaceutical developments for which molecular hybrid imaging diagnostics using PET/CT and PET/MRI are used for staging and treatment planning of a given disease (3–9). Many of the new radiotracers are being developed academically and tested at the interface between nuclear medicine and radiopharmacy and the linked medical disciplines benefiting from radiopharmaceutical applications. To be able to produce widely used radiotracers for everyday clinical care, in addition to e.g., specialized knowledge in the GMP-production of radiopharmaceuticals (10), regional access to a (medical) cyclotron is required in view of the short half-lives of clinically relevant radionuclides (11–13). Therefore, the demand for cyclotrons, which are a prerequisite for radionuclide production, has increased worldwide due to widespread application of PET examinations. A cyclotron is a specialized, large-scale device (14, 15) in which charged particles such as protons, deuterons or alpha particles can be accelerated to very high speeds in a circular path and directed on predominantly liquid and solid targets consisting of stable (enriched) isotopes converted into corresponding radionuclides by nuclear reactions (see Figure 1). The result is radioactive atoms that can be separated from the target material and radiochemically processed in synthesis modules (radiosynthesizers) for the automated production of tracers, which are formulated mainly into saline PET radiopharmaceutical injection solutions to image patients by intravenous injection of the radiopharmaceutical (15, 16). Common short-lived radionuclides used for PET imaging include e.g., ^18^F, ^68^Ga, ^11^C, ^13^N, ^15^O, ^44^Sc, ^64^Cu, ^89^Zr, ^86^Y and ^124^I (9, 17–21).

Since the last comprehensive publication with details on cyclotron infrastructure is more than 15 years old (22), our goal was to provide an academically-initiated update on the cyclotrons (location, number, type, etc.) operated for the production of radionuclides used for patient care in the German-speaking countries Germany (D), Austria (A) and Switzerland (CH). While the production of radionuclides and radiopharmaceuticals is comparable with other countries, differences are found within the regulatory framework (23, 24), in particular on a national level (25, 26). Yet, regulatory issues are not within the wider scope of this manuscript.

## Methods

For this overview, (medical) cyclotrons in the D-A-CH-countries were of interest, which are used for radionuclide-driven academic research and development as well as the production of radiopharmaceuticals for daily patient care. This included cyclotrons that are operated by medical and research institutions as well as by/with industrial partners. The focus was therefore on cyclotrons that have the ability to produce at least the most relevant radionuclides for nuclear medicine diagnostics and therapy and are actively in operation. Cyclotrons that still have an operating permit, but were e.g., “standby,” were not considered. The main characteristics of interest were the location of the cyclotron, the operating facility as well as the type, manufacturer and the (maximum) proton/particle energy of the cyclotron [with a view to the latter, the overview in (21), pp. 7–9, was used]. The data acquisition consisted of three steps:

- Firstly, publicly available information on the cyclotron infrastructure was investigated and compiled. This is in particular the written publications of the International Atomic Energy Agency (IAEA) (22, 27, 28) as well as the worldwide online-based “Cyclotron Master-List” (29) and “Database of Cyclotrons for Radionuclide Production” (30), which are based on work by the Radioisotope Products and Radiation Technology Section of the IAEA and provided by the member states and the operating facilities.
- Secondly, the information was updated and unclear and/or missing information was added by selective desktop research, e. g., on the websites of the departments / clinics of nuclear medicine or in academic publications with information details on the use of a cyclotron.
- Thirdly, the consolidated list was validated by members of the committee of the “Working Group Radiochemistry and Radiopharmacy” [AGRR, belonging to the German Society for Nuclear Medicine (DGN), see details under (31)], consisting of radiochemists and radiopharmacists from mainly academically-driven institutions within the D-A-CH countries, and the Radiopharmaceuticals Committee of the DGN.

With a view to the exploratory research goal, the results were evaluated descriptively and compiled in tables.

## Results

### Number of Operated Cyclotrons

As a result, 42 cyclotrons were identified that are currently being operated in the D-A-CH-countries for the production of radionuclides for nuclear medicine and radiopharmacy, 32 of them in Germany, 4 in Austria and 6 in Switzerland (see Table 1). In addition, the construction and commissioning of 6 additional cyclotrons is planned, 3 of them to be located in Germany and 3 to be located in Austria. The vast majority of the cyclotrons reported (67%) are operated by universities, university hospitals or research institutions in close proximity to a university hospital, less by/in cooperation with industrial partners (29%) or a non-academic clinic/PET-center (5%). This ratio between academia and industry operated cyclotrons might be owed to geographical reasons when European radiopharmaceutical infrastructure is compared to the one in the US (e.g., short vs. large distances), also differences in regulation play a role (23, 58).

**Table 1:** Overview of the cyclotrons operated for radionuclide production in the German-speaking D-A-CH-countries (as of December 2021).

**No**.	**Location**	**Organization/ Operator**	**Department/Clinic**	**Operator form^a^**	**Cyclotron (** **21** **)**			**Further references/ sample publications [besides (29, 30)]**
					**Manufacturer**	**Name/model/type**	**Proton energy (MeV)**	
**Germany (D)**								
1	Aachen	RWTH Aachen University	Department of Nuclear Medicine	U	GE Healthcare	PETtrace^TM^	16.5	
2	Bad Berka	Zentralklinik	Clinic for Nuclear Medicine	C	GE Healthcare	PETtrace^TM^	16.5	(32)
3	Bad	Heart and Diabetes Center	Institute for Radiology,	U	IBA	CYCLONE 18/9^TM^	16	(33)
4	Oeynhausen	North Rhine Westphalia, University Hospital, Ruhr University Bochum	Nuclear Medicine and Molecular Imaging	U	GE Healthcare	PETtrace 800^TM^	16.5	
5	Berlin	Life Radiopharma Berlin GmbH		I	GE Healthcare	PETtrace 880^TM^	16.5	
6		Diagnostic-Therapeutic Center (DTZ)	Center für Nuclear Medicine, DTZ radiochemistry	C	GE Healthcare	MINItrace 700^TM^	16	(34)
7	Bonn	Life Radiopharma Bonn GmbH		I	Siemens/CTI	RDS Eclipse 112^TM^	16	
8		Advanced Accelerator Applications (AAA)		I	GE Healthcare	PETtrace 800^TM^	16.5	
9	Dresden	Helmholtz-Zentrum Dresden-Rossendorf	Institute of Radiopharmaceutical Cancer Research	U	Advanced Cyclotron Systems (ACSI)	TR-Flex^TM^	16	(35, 36)
10	Essen	Essen University Hospital	Clinic for Nuklear Medicine,	U	IBA	Cyclone 18^TM^	16	(37)
11			Institute for Medical Radiation Physics	U	TCC	CV28^TM^	16	
12	Freiburg	EuroPET GmbH	on the premises of the Freiburg University Medical Center	I	GE Healthcare	PETtrace^TM^	16.5	
13	Hannover	Hannover Medical School (MHH)	Department of Nuclear Medicine	U	Siemens/CTI	RDS Eclipse^TM^	16	(38)
14	Jülich	Forschungszentrum Jülich	Institute of Neuroscience	U	GE Healthcare	PETtrace^TM^	16.5	(39, 40)
15		GmbH	and Medicine, Nuclear	U	Japan Steel Works	BC 1710^TM^	16	
16			Chemistry (INM-5)	U	IBA	Cyclone 30^TM^	16	
17	Karlsruhe,	ZAG Zyklotron AG		I	TCC	CP-42^TM^	16	(41)
18	Eggenstein-Leopoldshafen			I	Advanced Cyclotron Systems (ACSI)	TR-19/9^TM^	16	
19	Leipzig	University Hospital Leipzig	Department for Nuclear Medicine	U	GE Healthcare	PETtrace 860^TM^	16.5	(42)
20		Helmholtz-Zentrum Dresden-Rossendorf, research site Leipzig	Institute of Radiopharmaceutical Cancer Research	U	IBA	Cyclone 18^TM^	16	(43)
21	Magdeburg	Otto-von-Guericke-Universität Magdeburg, Medical Faculty		U	GE Healthcare	MINItrace 700s^TM^	16	(44)
22	Mainz	Johannes Gutenberg-University Mainz	Institute of Nuclear Chemistry	U	GE Healthcare	MINItrace 700s^TM^	16	(45)
23	Munich	Klinikum rechts der Isar Munich	Department of Nuclear Medicine	U	GE Healthcare	PETtrace 880^TM^	16.5	(46)
24		LMU Klinikum der Universität München^b^	Department of Nuclear Medicine	I	GE Healthcare	PETtrace 880^TM^	16.5	
25	Münster	University Hospital Münster	Department of Nuclear Medicine	U	Siemens/CTI	RDS-111^TM^	16	
26		Westfälische Wilhelms-Universität Münster (WWU)	Multiscale Imaging Center	U	IBA	Cyclone® KIUBE 18^TM^	16	(47)
27	Radeberg	ABX GmbH		I	GE Healthcare	MINItrace 700S^TM^	16	(4)
28	Regensburg	University Hospital Regensburg	Department of Nuclear Medicine	U	Siemens/CTI	RDS-111^TM^	16	
29	Rostock	Rostock University Medical Center	Department of Nuclear Medicine	U	GE Healthcare	MINItrace 700^TM^	16	(48)
30	Tübingen	University Hospital, Eberhard Karls University of Tübingen	Department of Preclinical Imaging and Radiopharmacy, Werner Siemens Imaging Center	U	GE Healthcare	PETtrace 890^TM^	16.5	(49)
31	Ulm	University Hospital Ulm	Department of Nuclear Medicine	U	GE Healthcare	PETtrace 860^TM^	16.5	(50, 51)
32	Würzburg	University Hospital of Würzburg	Department of Nuclear Medicine	U	GE Healthcare	PETtrace^TM^	16.5	(5, 52)
**Austria (A)**								
33	Klagenfurt	Argos Zyklotron Klagenfurt^c^		I	GE Healthcare	PETtrace^TM^	16.5	(53)
34	Linz	Argos Zyklotron Linz^c^		I	GE Healthcare	PETtrace^TM^	16.5	(53)
35	Seibersdorf	Seibersdorf Laboratories		I	GE Healthcare	PETtrace^TM^	16.5	(54)
36	Wien	Medical University of Vienna	Division of Nuclear Medicine	U	GE Healthcare	PETtrace 860^TM^	16.5	(55)
**Switzerland (CH)**								
37	Bern	SWAN Isotopen AG	located on the Campus of the University Hospital Bern	I	IBA	CYCLONE 18 Twin HC^TM^	16	
38	Genf	University Hospital of Geneva	Cyclotron Unit	U	IBA	Cyclone 18^TM^	16	
39	Villigen	Paul Scherrer Institute (PSI)	Center for Radiopharmaceutical Sciences	U	PSI	Cockcroft Walton^TM,^^d^	16	(56)
40	Zürich	ETH Zürich	Swiss Federal Institute of Technology Zurich	U	IBA	Cyclone 18^TM^	16	
41	Zürich/Schlieren	University Hospital Zürich	Schlieren Laboratory, Oncology Clinic (Wagi)	U	GE Healthcare	PETtrace^TM^	16.5	(57)
42	Zürich	University Hospital Zürich	PET Center	U	GE Healthcare	PETtrace^TM^	16.5	(57)

a *U = University, University Clinic, Research Organization; C = Clinic, Medical Care/(Non-University) PET/Theranostics-Center; I = Commercial Provider, Cyclotron Facility Operated by/With Industrial Partner*.

b *In Cooperation With AAA München GmbH^TM^ (Public-Private Partnership, PPP)*.

c *Operated for IASON™*.

d *Cockcroft Walton (870 KeV)/“Injector 2” Zyklotron*.

### Cyclotron Manufacturer and Type

Most of the actively operated cyclotrons reported were manufactured by GE Healthcare (57%) or Ion Beam Applications (IBA) (16%) (cf. this and the following Table 2). Other manufacturers play a minor role (altogether 24%). While cyclotrons from several manufacturers can be found in Germany and Switzerland, the cyclotrons listed for Austria are solely by GE Healthcare.

**Table 2:** Cyclotrons currently operated for radionuclide production in German-speaking D-A-CH-countries countries according to manufacturer and proton energy (MeV), as of December 2021.

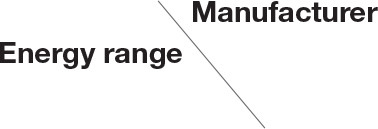	**GE Healthcare**		**IBA**		**Other (ACSI, TCC, Siemens/CTI, etc.)**		**Total**	
	**Absolute (*n*)**	**Relative**	**Absolute (*n*)**	**Relative**	**Absolute (*n*)**	**Relative**	**Absolute (*n*)**	**Relative**
up to 10 MeV	16	12%					**5**	12%
11–20 MeV	16	45%	7	17%	6	14%	**32**	76%
20–30 MeV			1	2%	2	5%	**3**	7%
> 30 MeV					2	5%	**2**	5%
**Total**	**24**	57%	**8**	19%	**10**	24%	**42**	100%

*Values in bold represent totals*.

With regard to the proton energy of the cyclotrons, it is noticeable that most of the reported cyclotrons achieve 11–20 MeV (76%), followed by up to 10 MeV (12%). The cyclotron with the highest MeV proton beam is located at the Paul Scherrer Institute (PSI) in Switzerland (72 MeV), the cyclotrons with the lowest MeV proton beam are part of the GE Healthcare MINItrace^TM^ series (several locations, e. g., Berlin, Mainz, Rostock).

## Discussion and Conclusions

The development and production of radiopharmaceuticals e.g., for non-invasive molecular PET/CT- and PET/MR imaging and therewith the need to produce cyclotron-based radiopharmaceuticals onsite have become more and more important. Against this background, we wanted to provide an updated overview of the (medical) cyclotrons currently operated for nuclear medicine and radiopharmacy in the D-A-CH countries. The publication thus builds on essential publications and work on cyclotron infrastructure-related aspects [e.g., (21, 22, 27, 59)] and is intended to supplement these as an update for the D-A-CH region. In the following, we discuss the current status and trends, address the importance of (research) centers specialized in the cyclotron-based development and production of radiopharmaceuticals in these countries and present limitations of our work.

### Development, Current Status and Trends

The number of actively operated cyclotrons in the D-A-CH-countries has increased significantly with in total *n* = 42 since the publications by the IAEA in 2006 [*n* = 28, (22)] and 1998 [*n* = 23, (27)]. A main reason for this is likely to be the increasing demand for nuclear medicine diagnostic and therapeutic procedures for which cyclotron-based radiopharmaceuticals are used. Consequently, the number of cyclotron installations is also increasing. Cyclotron sites in Germany, that went recently into operation, include e.g., Rostock, Magdeburg (44) or Mainz (45). In other German locations such as Bad Berka or Dresden-Rossendorf (35), new cyclotrons have been recently installed, which are replacing old accelerators. In addition, the construction plans for cyclotron locations such as health facilities in the locations of Essen (60) and Mannheim (61) for Germany and Vienna and Graz for Austria have already been announced. In contrast, it should also be noted that several cyclotrons have been taken out of service or were decommissioned at several locations. Examples of the latter are in Germany e. g., the cyclotrons at the German Cancer Research Center (DKFZ) in Heidelberg (out of operation) or at the Max Planck Institute for Neurological Research (now: MPI for Metabolic Research) in Cologne (decommissioned).

The market leader in the D-A-CH countries is GE Healthcare (57%), with the PETtrace^TM^ cyclotron series in particular being put into operation about 30 years ago. In 2018, the company GE Healthcare announced that it had installed its 400^th^ medical cyclotron worldwide (62), according to which around 6 percent of the cyclotrons installed by the manufacturer can be found in the D-A-CH-countries. In addition, it becomes clear, that many of the (comparatively newly installed) cyclotrons have a proton beam of up to 18 MeV. With regard to the production of radionuclides for patient care, 18 MeV are ideally suited to produce the most common radionuclides for PET imaging such as ^18^F, ^11^C, ^64^Cu, ^68^Ga and ^89^Zr. Systems with higher beam energies, which enable the production of further SPECT and therapeutic radionuclides like ^123^I and ^67^Cu [e.g., the Jülich Cyclone® 30 XP (39)], on the other hand, were installed less often or even decommissioned. Due to the ongoing rise of applications with the well-known beta^−^particle emitter ^177^Lu, but also with alpha particle emitters such as ^211^At and ^225^Ac, the timing is good to invest in infrastructure for the production of alternative beta^−^ and alpha particle emitters to add on the capacities of the predominantly clinically used radionuclides ^131^I, ^177^Lu and ^90^Y. For that purpose different accelerators with flexibilities in different nuclear reactions can be used to realize nuclear reactions such as (p, x), (d, x), (α, x) and also (γ, x), (n, x) as subsequent processes.

The latter development is important because universities, university hospitals and research institutions in the D-A-CH countries have been relatively successful in the academically-driven development and medical translation of novel radiopharmaceuticals in general and cyclotron-based tracers in particular for nuclear medicine applications in recent years. Examples for Germany include ^18^F-labeled tracers like [^18^F]PSMA-1007 (4, 63), 2-[^18^F]fluoroethyl-l-tyrosine ([^18^F]FET) (64), [^18^F]JK-PSMA-7 (65) or [^18^F]flortaucipir (Tauvid™) (66). In view of the increasing (planned) new approval of cyclotron-based radiotracers and further developments in clinical studies (2, 8, 67) as well as the existing production requirements for 2-[^18^F]fluoro-2-deoxy-D-glucose ([^18^F]FDG) in particular (68) it is important from the patient's point of view that the radiopharmaceutical infrastructures required for innovative nuclear medicine diagnostics and therapy procedures grows accordingly. In addition, the authors point out that with the increasing number of innovative applications in the field of endoradionuclide therapy, fueled by, among others, [^177^Lu]Lu-DOTA-TATE (Lutathera®) and [^177^Lu]Lu-PSMA-617, there is simultaneously more demand for accompanying diagnostic procedures or prestaging and monitoring diagnostics with corresponding ^18^F, ^68^Ga or ^64^Cu, ^89^Zr, ^44^Sc labeled radiotracers. This can already be seen and we expect further professionalization on the academic and industrial side through the installation of further medical cyclotrons. In addition, it can be seen that both academic institutions and industrial companies are investing heavily in the establishment of further therapeutic radionuclides beyond ^177^Lu. Examples include ^225^Ac (alpha), ^67^Cu (beta^−^), ^161^Tb (beta^−^ & Auger), ^212^Pb (beta^−^ & alpha). The authors therefore assume that the need for cyclotron infrastructure in the D-A-CH region will continue to grow. How important the early consideration of these infrastructure-related aspects can be for patient care is shown, for example, by the result of a current calculation, according to which that the capacity of nuclear medicine therapy beds in Germany is likely to be very well utilized with a prospectively approved therapeutic agent for [^177^Lu]Lu-PSMA-RLT, and could even reach its limits in some German federal states (69). The present data can thus serve as a reference point for future decision-makers on capacity planning issues related to cyclotron infrastructure in the D-A-CH region. Further future research could supplement this overview, e.g., with a view to radionuclide-specific aspects or other structural parameters such as the personnel required for cyclotron operation (70).

### Organizational Trends and Operational Aspects

In the last years, molecular imaging was additionally boosted by the introduction of the theranostic concept, which offers hospitals various opportunities to improve patient care in the field of nuclear medicine (71). From a business point of view, clinics have to consider both the radiopharmaceutical-clinical and infrastructure-cost-related aspects of various radiopharmaceuticals for patient care. Firstly, it should be noted that the planned construction and operation of a cyclotron is associated with high costs. In addition to the procurement costs for the cyclotron (starting at around EUR 1–3 million), there are additional costs for the construction of the building as well as the radiochemical laboratories, radiopharmaceutical GMP production site, hot cells, synthesis units and specialized equipment (72). For the cyclotron operation there are ongoing costs for personnel and material resources. Against this background, it is not surprising that some sites in the D-A-CH countries produce medical radionuclides for several facilities. Examples of this “satellite concept” are in Germany the Jülich site, whose cyclotrons supply both the internal department and medical facilities in the Aachen, Düsseldorf and Cologne/Bonn regions, or the Heart and Diabetes Center in Bad Oeynhausen, whose cyclotrons supply clinics in East Westphalia and throughout northwest Germany. The relatively newly built cyclotron in Magdeburg (44) is also planned to supply several facilities. The authors point out, that in principle with a consequent use of the “satellite concept” a nearly area-wide supply with cyclotron produced medical radionuclides in the D-A-CH area could be realized. However with a view to the implementation of such a “satellite concept,” it should be pointed out that some countries interpret the European rules regarding a need for a marketing authorization as laid down in Directive 2001/83/EC applicable to kits, generators and so called radionuclide precursors very strictly (73). According to German national law, for example, a marketing authorization is needed not only for radionuclide precursors that are used in subsequent kit-like preparations but as well for radionuclides that are used as starting materials in complex manufacturing of radiopharmaceuticals for human use [cf. (74) on this and the current regulatory status for Germany]. Generally, current regulation for radiopharmaceuticals on national levels within Europe is diverse (25, 26).

In addition, universities, university hospitals and research organizations should carefully check which of the radionuclides they aim to produce using the cyclotron (and thus which cyclotron needs to be procured). This can first be illustrated using the example of prostate cancer diagnostics using the PSMA radioligand approach: For the production of ^68^Ga-labeled PSMA-radioligands for example, a ^68^Ge/^68^Ga-generator is primarily used whereas you need a highly regulated cyclotron infrastructure for the production of ^18^F-labeled tracers. Advantages of the latter include a higher production yield and a longer half-life compared to ^68^Ga-labeled ligands (110 vs. 68 min). It is therefore possible to transport ^18^F-tracers over a certain distance, for instance, to external hospitals or application sites for the PET scan (75). Due to the different properties, hospitals with a rather small number of prostate cancer patients and/or without a cyclotron on-site could opt for ^68^Ga-labeled PSMA-radioligands produced based on a ^68^Ge/^68^Ga-generator, because the purchase and operation of a cyclotron is associated with comparatively high financial expenditure with small economies of scale. Clinics, with a high number of patients needed to treat, and a cyclotron on-site, on the other hand, will generally use the ^18^F-labeled ligands as their PSMA-tracer of choice amongst others because of the decreasing marginal costs. As an alternative, they could also opt for the production of ^68^Ga-tracers via the cyclotron if there is only a need for a small amount for in-house use (8, 76).

Finally, it becomes apparent that in recent years more and more cyclotrons are operated by and/or in cooperation with private companies and investors, also potentially driven by major drug companies rediscovering the clinical value of radiopharmaceuticals (1, 77, 78). For logistical reasons, some facilities such as those in Freiburg and Munich in Germany or Bern in Switzerland are located in the immediate vicinity or directly on the campus of a university hospital. The business idea behind it: through the cooperation in the manufacture of cyclotron-based PET-radiopharmaceuticals, competencies for the development of novel radiopharmaceuticals can be pooled and the utilization of production capacities can be optimized, both bringing down costs and producing efficiency gains. The basis for this is usually a cooperation agreement between the institutions.

### Limitations

In view of the increasing number of radiopharmaceuticals developed and used in everyday clinical care, our aim was to provide an updated overview of (medical) cyclotrons that are currently being used for the production of medical radionuclides in the D-A-CH-countries. Devices that were currently not used in daily operation or were decommissioned (e.g., cyclotrons in the cities of Cologne, Erlangen or Heidelberg) were not considered. From a methodological point of view, it should be pointed out that the data collection and compilation of the operated cyclotrons is based on publicly accessible secondary information only. It could therefore be assumed that the actual number of cyclotrons in use for the production of radionuclides in the D-A-CH-region may be slightly underestimated. This could be the case in particular for cyclotrons operated by industrial partners. The reason for this is, that universities or academic institutions often report in more detail about the cyclotron infrastructure at a location [see e.g., (35)] than commercial providers. This also applies to new acquisitions or planned installations (60, 61). Finally, it should be pointed out that the cyclotron infrastructure is constantly changing due to the age of the systems and health policy and economic decisions. We can therefore offer an updated overview of the current status, but cannot guarantee completeness, so that the cyclotron overview must be updated at regular intervals.

## Summary

- In recent years, innovative nuclear medicine methods for non-invasive molecular hybrid imaging diagnostics using radiopharmaceuticals have been developed and established in patient care.
- In order to be able to produce the necessary radiopharmaceuticals with often short physical half-lives in large quantities the access to a cyclotron is required.
- We are presenting an updated overview of the cyclotron infrastructure for the German speaking D-A-CH-countries.
- Overall, it is demonstrated that in recent years more cyclotrons have been installed to supply several (research) facilities and are more often operated in cooperation with or by industrial partners.
- With a view to medical-technical progress as well as political and regulatory developments, a regularly updated overview on the cyclotron infrastructure would be desirable.

## Author's Note

The present article is based on a secondary data analysis with research status December 2021. However, the authors are aware that the landscape of (medical) cyclotrons operated for nuclear medicine and radiopharmacy is continuously developing and changing over time. Therefore, if you have updated information on site-specific changes to the cyclotron infrastructure in Germany, Austria and Switzerland, please let us know by emailing us at agrr@nuklearmedizin.de.

## Data Availability Statement

The original contributions presented in the study are included in the article/supplementary material, further inquiries can be directed to the corresponding author/s.

## Ethics Statement

Ethical review and approval was not required for the study on human participants, in accordance with the local legislation and institutional requirements.

## Author Contributions

CZ and KK developed the principal idea of the article/research method, conducted a search of the literature, wrote, reviewed, edited, and formatted the draft of the manuscript. JE, MP, FG, TR, GR, TK, CS, BN, OK, MM, WW, and RS contributed to the drafting and critically revised the manuscript for important intellectual content. All authors have read and agreed to the published version of the manuscript. All authors contributed to the consolidation and validation of the data on the cyclotron landscape.

## Conflict of Interest

The authors declare that the research was conducted in the absence of any commercial or financial relationships that could be construed as a potential conflict of interest.

## Publisher's Note

All claims expressed in this article are solely those of the authors and do not necessarily represent those of their affiliated organizations, or those of the publisher, the editors and the reviewers. Any product that may be evaluated in this article, or claim that may be made by its manufacturer, is not guaranteed or endorsed by the publisher.
